# Supplementary material for: A Likelihood Approach for Real-Time Calibration of Stochastic Compartmental Epidemic Models
Source: PLoS Comput Biol. 2017 Jan 17;13(1):e1005257. doi: 10.1371/journal.pcbi.1005257 (PMC5240920; doi:10.1371/journal.pcbi.1005257)
Supplement: S1 File — (TAR.GZ) [file pcbi.1005257.s014.tar.gz › HSPH_Online-SI-Revision/Additions/MSS11/n100-extreme-SEITR_noisy/n100-extreme-SEITR_noisy_NEW2_table8.pdf]

1	newly 3 weeks spec	50.75	599.46	{565., 633.}	1081.2	1.07191	newly 3 weeks cumul	584.434	2542.62	{2495., 2597.}	335.057	0.638485	newly 5 weeks spec	42.6203	315.63	{290., 340.}	640.562	0.868632	newly 5 weeks cumul	687.444	3293.01	{3228., 3352.}	379.022	0.680311	newly 42 weeks cumul	3753.74	4124.	{4038., 4194.}	9.86372	0.0408053
2	newly 3 weeks spec	102.	129.1	{114., 144.}	26.6863	0.100893	newly 3 weeks cumul	593.	714.53	{684., 749.}	20.4941	0.0806891	newly 5 weeks spec	36.	45.88	{36., 55.}	29.7222	0.109456	newly 5 weeks cumul	673.	836.32	{795., 875.}	24.2675	0.0940836	newly 50 weeks cumul	712.	901.71	{851., 939.}	26.6447	0.102292
3	newly 3 weeks spec	169.	227.44	{209., 247.}	34.5799	0.128025	newly 3 weeks cumul	920.	1177.7	{1131., 1215.}	28.0109	0.107095	newly 5 weeks spec	52.	87.17	{74., 102.}	67.6346	0.221024	newly 5 weeks cumul	1072.	1404.11	{1353., 1449.}	30.9804	0.117065	newly 52 weeks cumul	1137.	1548.02	{1493., 1598.}	36.1495	0.13387
4	newly 3 weeks spec	137.	153.71	{136., 171.}	13.4526	0.053839	newly 3 weeks cumul	790.	841.78	{802., 877.}	6.58481	0.0274521	newly 5 weeks spec	41.	55.65	{46., 67.}	36.5122	0.130686	newly 5 weeks cumul	909.	990.71	{952., 1032.}	9.0088	0.0372378	newly 50 weeks cumul	985.	1073.97	{1030., 1125.}	9.03249	0.0373028
5	newly 3 weeks spec	34.	26.11	{20., 33.}	24.6765	0.129195	newly 3 weeks cumul	171.	174.66	{157., 190.}	6.18713	0.026465	newly 5 weeks spec	11.	7.01	{4., 11.}	39.1818	0.264191	newly 5 weeks cumul	195.	195.69	{177., 213.}	6.06667	0.0263805	newly 52 weeks cumul	201.	203.24	{182., 223.}	6.30846	0.0271944
6	newly 3 weeks spec	5.	84.1	{73., 97.}	1582.	1.22318	newly 3 weeks cumul	60.	472.9	{448., 498.}	688.167	0.896206	newly 5 weeks spec	2.	27.73	{20., 35.}	1286.5	1.13202	newly 5 weeks cumul	63.	549.02	{521., 575.}	771.46	0.93987	newly 51 weeks cumul	64.	585.32	{552., 619.}	814.563	0.960759
7	newly 3 weeks spec	469.	656.48	{623., 690.}	39.9744	0.145726	newly 3 weeks cumul	2142.	2748.38	{2690., 2808.}	28.3091	0.108194	newly 5 weeks spec	221.	359.01	{333., 381.}	62.448	0.210178	newly 5 weeks cumul	2717.	3595.11	{3535., 3665.}	32.3191	0.121582	newly 43 weeks cumul	3226.	4584.38	{4502., 4658.}	42.1073	0.152579
8	newly 3 weeks spec	225.	119.	{105., 133.}	47.1111	0.27876	newly 3 weeks cumul	1088.	657.94	{622., 694.}	39.5276	0.218784	newly 5 weeks spec	80.	43.07	{34., 54.}	46.1625	0.27594	newly 5 weeks cumul	1321.	771.84	{732., 808.}	41.5715	0.233683	newly 50 weeks cumul	1472.	831.39	{787., 869.}	43.5197	0.248423
9	newly 3 weeks spec	19.	25.58	{18., 32.}	37.7895	0.133975	newly 3 weeks cumul	167.	171.88	{155., 188.}	5.94012	0.0251859	newly 5 weeks spec	5.	6.56	{3., 10.}	46.8	0.178745	newly 5 weeks cumul	179.	191.97	{176., 211.}	8.53073	0.0351075	newly 52 weeks cumul	188.	198.89	{182., 217.}	7.53723	0.0311606
10	newly 3 weeks spec	613.	375.16	{350., 403.}	38.7993	0.213874	newly 3 weeks cumul	2661.	1794.89	{1746., 1840.}	32.5483	0.171093	newly 5 weeks spec	260.	163.93	{143., 183.}	36.95	0.201963	newly 5 weeks cumul	3324.	2208.76	{2158., 2262.}	33.5511	0.177584	newly 48 weeks cumul	3949.	2533.16	{2473., 2593.}	35.8531	0.192899
11	newly 3 weeks spec	448.	337.95	{308., 359.}	24.5647	0.123092	newly 3 weeks cumul	2137.	1631.53	{1591., 1675.}	23.6533	0.117306	newly 5 weeks spec	237.	147.85	{132., 164.}	37.616	0.20689	newly 5 weeks cumul	2689.	2001.91	{1946., 2058.}	25.5519	0.128244	newly 47 weeks cumul	3221.	2280.03	{2213., 2350.}	29.2136	0.150179
12	newly 3 weeks spec	209.	327.54	{306., 351.}	56.7177	0.194513	newly 3 weeks cumul	1086.	1555.07	{1511., 1602.}	43.1924	0.155818	newly 5 weeks spec	68.	145.53	{127., 162.}	114.015	0.328636	newly 5 weeks cumul	1294.	1920.8	{1873., 1979.}	48.4389	0.171461	newly 48 weeks cumul	1442.	2213.86	{2161., 2273.}	53.527	0.186098
13	newly 3 weeks spec	300.	240.27	{222., 259.}	19.91	0.0973896	newly 3 weeks cumul	1508.	1194.65	{1150., 1234.}	20.7792	0.101311	newly 5 weeks spec	131.	97.8	{85., 111.}	25.3435	0.129481	newly 5 weeks cumul	1818.	1444.56	{1394., 1486.}	20.5413	0.0999813	newly 49 weeks cumul	2109.	1617.16	{1573., 1661.}	23.321	0.115437
14	newly 3 weeks spec	681.	816.46	{779., 850.}	19.8913	0.0785443	newly 3 weeks cumul	2981.	3384.84	{3314., 3450.}	13.5471	0.0551238	newly 5 weeks spec	331.	447.28	{420., 475.}	35.1299	0.130222	newly 5 weeks cumul	3718.	4431.36	{4363., 4502.}	19.1867	0.0761867	newly 42 weeks cumul	4474.	5675.65	{5571., 5768.}	26.8585	0.103277
15	newly 3 weeks spec	238.	658.06	{621., 688.}	176.496	0.441356	newly 3 weeks cumul	1233.	2756.34	{2699., 2811.}	123.547	0.349312	newly 5 weeks spec	100.	354.04	{330., 384.}	254.04	0.548336	newly 5 weeks cumul	1479.	3594.88	{3525., 3659.}	143.062	0.385667	newly 43 weeks cumul	1667.	4580.01	{4490., 4658.}	174.746	0.438889
16	newly 3 weeks spec	233.	688.89	{646., 721.}	195.661	0.470428	newly 3 weeks cumul	1269.	2962.37	{2898., 3018.}	133.441	0.368125	newly 5 weeks spec	91.	356.3	{333., 377.}	291.538	0.592201	newly 5 weeks cumul	1497.	3815.86	{3754., 3879.}	154.9	0.406336	newly 46 weeks cumul	1647.	4738.57	{4653., 4815.}	187.709	0.458913
17	newly 3 weeks spec	1.	18.77	{12., 25.}	1777.	1.25517	newly 3 weeks cumul	62.	144.23	{124., 164.}	132.629	0.364123	newly 5 weeks spec	0.1	4.48	{1., 8.}	4394.	1.48442	newly 5 weeks cumul	64.	158.24	{132., 184.}	147.25	0.390007	newly 52 weeks cumul	64.	162.38	{135., 192.}	153.719	0.400903
18	newly 3 weeks spec	93.	84.42	{72., 97.}	11.2473	0.0535175	newly 3 weeks cumul	605.	485.56	{463., 512.}	19.7421	0.0958321	newly 5 weeks spec	53.	27.31	{20., 36.}	48.4717	0.300029	newly 5 weeks cumul	722.	561.63	{535., 587.}	22.2119	0.109398	newly 51 weeks cumul	795.	597.1	{569., 625.}	24.8931	0.124642
19	newly 3 weeks spec	465.	686.56	{653., 731.}	47.6473	0.168833	newly 3 weeks cumul	2284.	3045.46	{2977., 3105.}	33.3389	0.124907	newly 5 weeks spec	217.	337.99	{315., 364.}	55.7558	0.191812	newly 5 weeks cumul	2812.	3866.81	{3793., 3931.}	37.511	0.138298	newly 46 weeks cumul	3336.	4653.63	{4575., 4731.}	39.4973	0.144523
20	newly 3 weeks spec	75.	83.16	{70., 98.}	13.9733	0.0557989	newly 3 weeks cumul	401.	473.18	{445., 506.}	18.	0.0714225	newly 5 weeks spec	14.	26.67	{21., 33.}	90.5	0.27125	newly 5 weeks cumul	459.	548.57	{517., 578.}	19.5142	0.0770422	newly 51 weeks cumul	486.	586.6	{558., 619.}	20.6996	0.0813537
21	newly 3 weeks spec	38.	27.34	{19., 35.}	28.4211	0.155691	newly 3 weeks cumul	270.	177.16	{156., 194.}	34.3852	0.184322	newly 5 weeks spec	12.	6.61	{3., 10.}	45.75	0.300283	newly 5 weeks cumul	303.	196.99	{176., 216.}	34.9868	0.188392	newly 52 weeks cumul	316.	205.11	{183., 223.}	35.0918	0.189041
22	newly 3 weeks spec	162.	323.73	{296., 344.}	99.8333	0.300008	newly 3 weeks cumul	806.	1549.26	{1513., 1587.}	92.2159	0.283718	newly 5 weeks spec	58.	144.51	{131., 159.}	149.155	0.394936	newly 5 weeks cumul	951.	1915.5	{1869., 1966.}	101.42	0.304018	newly 48 weeks cumul	1042.	2202.48	{2134., 2262.}	111.37	0.324939
23	newly 3 weeks spec	22.	49.42	{37., 63.}	124.636	0.342178	newly 3 weeks cumul	142.	285.85	{263., 311.}	101.303	0.302901	newly 5 weeks spec	3.	15.35	{10., 22.}	411.667	0.690239	newly 5 weeks cumul	156.	327.96	{296., 356.}	110.231	0.321673	newly 49 weeks cumul	161.	346.54	{311., 379.}	115.242	0.331767
24	newly 3 weeks spec	51.	100.69	{88., 113.}	97.4314	0.293242	newly 3 weeks cumul	312.	567.7	{535., 596.}	81.9551	0.259599	newly 5 weeks spec	15.	32.85	{24., 42.}	119.	0.330568	newly 5 weeks cumul	360.	659.04	{626., 689.}	83.0667	0.262275	newly 51 weeks cumul	369.	705.17	{668., 737.}	91.103	0.280932
25	newly 3 weeks spec	160.	334.39	{310., 362.}	108.994	0.319407	newly 3 weeks cumul	871.	1622.79	{1570., 1667.}	86.3134	0.270117	newly 5 weeks spec	60.	147.1	{129., 159.}	145.167	0.387752	newly 5 weeks cumul	1024.	1993.82	{1932., 2045.}	94.709	0.289288	newly 47 weeks cumul	1086.	2269.82	{2197., 2337.}	109.007	0.320038
26	newly 3 weeks spec	96.	405.78	{303., 505.}	322.688	0.616652	newly 3 weeks cumul	682.	1853.15	{1510., 2184.}	171.723	0.428087	newly 5 weeks spec	33.	192.59	{137., 248.}	483.606	0.753142	newly 5 weeks cumul	784.	2329.89	{1873., 2764.}	197.18	0.466158	newly 45 weeks cumul	878.	2757.67	{2167., 3332.}	214.085	0.488541
27	newly 3 weeks spec	572.	485.77	{453., 517.}	15.0752	0.0714917	newly 3 weeks cumul	2438.	2149.65	{2090., 2198.}	11.8273	0.0547382	newly 5 weeks spec	284.	240.55	{222., 261.}	15.2993	0.0730351	newly 5 weeks cumul	3101.	2729.81	{2664., 2787.}	11.97	0.055434	newly 45 weeks cumul	3756.	3284.11	{3208., 3351.}	12.5636	0.0583725
28	newly 3 weeks spec	8.	55.31	{45., 67.}	591.375	0.83433	newly 3 weeks cumul	54.	342.34	{318., 365.}	533.963	0.801369	newly 5 weeks spec	1.	16.78	{11., 24.}	1578.	1.20847	newly 5 weeks cumul	61.	390.13	{365., 421.}	539.557	0.805162	newly 52 weeks cumul	63.	411.08	{387., 445.}	552.508	0.813835
29	newly 3 weeks spec	378.	349.18	{326., 372.}	7.95238	0.0365439	newly 3 weeks cumul	1806.	1705.86	{1669., 1744.}	5.54485	0.024842	newly 5 weeks spec	145.	151.37	{136., 165.}	7.12414	0.0298501	newly 5 weeks cumul	2196.	2088.87	{2038., 2141.}	4.87842	0.0217949	newly 49 weeks cumul	2473.	2372.45	{2306., 2433.}	4.10877	0.0182984
30	newly 3 weeks spec	146.	143.66	{124., 161.}	7.21918	0.0320789	newly 3 weeks cumul	831.	823.26	{791., 854.}	2.65945	0.0116638	newly 5 weeks spec	47.	47.84	{37., 58.}	12.766	0.0557026	newly 5 weeks cumul	975.	952.2	{908., 994.}	3.14667	0.0139593	newly 52 weeks cumul	1051.	1017.3	{973., 1060.}	3.59277	0.0160244
31	newly 3 weeks spec	292.	583.77	{511., 629.}	99.9212	0.299609	newly 3 weeks cumul	1423.	2486.95	{2233., 2597.}	74.7681	0.241816	newly 5 weeks spec	119.	303.77	{258., 332.}	155.269	0.405294	newly 5 weeks cumul	1719.	3215.52	{2850., 3352.}	87.0576	0.271261	newly 42 weeks cumul	1955.	4008.67	{3508., 4209.}	105.047	0.310986
32	newly 3 weeks spec	532.	556.53	{521., 594.}	5.28759	0.0221236	newly 3 weeks cumul	2380.	2462.14	{2406., 2513.}	3.5	0.0148835	newly 5 weeks spec	254.	272.35	{251., 295.}	8.14567	0.0335325	newly 5 weeks cumul	3005.	3122.71	{3068., 3177.}	3.91714	0.0166369	newly 48 weeks cumul	3596.	3750.69	{3670., 3824.}	4.30172	0.0182344
33	newly 3 weeks spec	65.	26.49	{18., 33.}	59.2462	0.400031	newly 3 weeks cumul	390.	175.05	{156., 191.}	55.1154	0.349088	newly 5 weeks spec	28.	7.72	{4., 12.}	72.4286	0.617497	newly 5 weeks cumul	468.	196.31	{175., 213.}	58.0534	0.378424	newly 52 weeks cumul	508.	204.33	{184., 224.}	59.7776	0.39682
34	newly 3 weeks spec	107.	230.27	{206., 248.}	115.206	0.331763	newly 3 weeks cumul	548.	1117.62	{1077., 1154.}	103.945	0.309375	newly 5 weeks spec	35.	98.2	{84., 113.}	180.571	0.44533	newly 5 weeks cumul	648.	1364.35	{1317., 1407.}	110.548	0.323212	newly 48 weeks cumul	692.	1549.65	{1495., 1599.}	123.938	0.349984
35	newly 3 weeks spec	10.	26.23	{18., 35.}	162.3	0.405828	newly 3 weeks cumul	81.	172.84	{152., 190.}	113.383	0.327619	newly 5 weeks spec	0.1	6.57	{3., 10.}	6470.	1.77539	newly 5 weeks cumul	82.	192.59	{171., 212.}	134.866	0.369407	newly 52 weeks cumul	82.	200.45	{180., 222.}	144.451	0.38675
36	newly 3 weeks spec	587.	323.91	{301., 347.}	44.8194	0.258893	newly 3 weeks cumul	2638.	1623.01	{1585., 1663.}	38.4757	0.211036	newly 5 weeks spec	275.	134.4	{116., 152.}	51.1273	0.313131	newly 5 weeks cumul	3306.	1971.61	{1926., 2018.}	40.3627	0.224556	newly 48 weeks cumul	3908.	2217.66	{2159., 2278.}	43.2533	0.246157
37	newly 3 weeks spec	138.	239.34	{220., 261.}	73.4348	0.238298	newly 3 weeks cumul	721.	1196.	{1161., 1234.}	65.8807	0.219654	newly 5 weeks spec	53.	97.87	{84., 109.}	84.6604	0.263988	newly 5 weeks cumul	854.	1444.78	{1405., 1483.}	69.178	0.228231	newly 49 weeks cumul	922.	1620.83	{1575., 1675.}	75.795	0.244889
38	newly 3 weeks spec	103.	277.96	{257., 299.}	169.864	0.430257	newly 3 weeks cumul	598.	1331.25	{1296., 1374.}	122.617	0.347452	newly 5 weeks spec	29.	121.7	{104., 138.}	319.655	0.619937	newly 5 weeks cumul	693.	1636.67	{1596., 1695.}	136.172	0.373121	newly 46 weeks cumul	752.	1878.57	{1815., 1943.}	149.81	0.39746
39	newly 3 weeks spec	188.	264.94	{247., 280.}	40.9255	0.148334	newly 3 weeks cumul	946.	1315.38	{1284., 1344.}	39.0465	0.14307	newly 5 weeks spec	61.	108.57	{91., 123.}	77.9836	0.247502	newly 5 weeks cumul	1112.	1593.11	{1544., 1638.}	43.2653	0.156038	newly 48 weeks cumul	1224.	1788.35	{1730., 1837.}	46.107	0.164549
40	newly 3 weeks spec	395.	270.51	{248., 289.}	31.5165	0.165155	newly 3 weeks cumul	1868.	1303.47	{1257., 1344.}	30.2211	0.156415	newly 5 weeks spec	218.	116.52	{103., 130.}	46.5505	0.274174	newly 5 weeks cumul	2391.	1598.73	{1544., 1638.}	33.1355	0.174916	newly 49 weeks cumul	2892.	1821.46	{1767., 1868.}	37.0173	0.200882
41	newly 3 weeks spec	12.	20.65	{13., 28.}	74.9167	0.230823	newly 3 weeks cumul	121.	147.29	{126., 167.}	22.2231	0.0849078	newly 5 weeks spec	2.	4.45	{1., 9.}	143.5	0.399799	newly 5 weeks cumul	135.	161.92	{138., 185.}	20.7852	0.079743	newly 52 weeks cumul	137.	166.1	{140., 190.}	22.	0.0840173
42	newly 3 weeks spec	675.	820.92	{789., 852.}	21.6178	0.0847524	newly 3 weeks cumul	2966.	3409.88	{3355., 3479.}	14.9656	0.060526	newly 5 weeks spec	340.	449.78	{423., 476.}	32.2882	0.121079	newly 5 weeks cumul	3794.	4471.03	{4410., 4539.}	17.8448	0.0712843	newly 43 weeks cumul	4690.	5751.76	{5663., 5844.}	22.6388	0.0886018
43	newly 3 weeks spec	545.	374.	{348., 400.}	31.3761	0.1641	newly 3 weeks cumul	2315.	1785.57	{1744., 1831.}	22.8695	0.112859	newly 5 weeks spec	245.	164.82	{147., 180.}	32.7265	0.173452	newly 5 weeks cumul	2918.	2199.99	{2153., 2250.}	24.6062	0.122726	newly 48 weeks cumul	3370.	2521.57	{2460., 2585.}	25.176	0.126034
44	newly 3 weeks spec	215.	261.71	{239., 284.}	21.7256	0.0845862	newly 3 weeks cumul	1211.	1316.19	{1277., 1360.}	8.68621	0.0360525	newly 5 weeks spec	93.	107.77	{91., 120.}	17.043	0.0669131	newly 5 weeks cumul	1475.	1594.01	{1549., 1645.}	8.06847	0.0335875	newly 48 weeks cumul	1690.	1790.61	{1746., 1851.}	6.01124	0.0252441
45	newly 3 weeks spec	201.	119.36	{103., 136.}	40.6169	0.228782	newly 3 weeks cumul	1070.	656.68	{622., 690.}	38.628	0.212331	newly 5 weeks spec	90.	42.38	{33., 51.}	52.9111	0.333244	newly 5 weeks cumul	1275.	769.14	{732., 801.}	39.6753	0.219796	newly 50 weeks cumul	1419.	830.99	{787., 869.}	41.4383	0.232707
46	newly 3 weeks spec	138.	227.21	{205., 246.}	64.6449	0.215381	newly 3 weeks cumul	722.	1179.52	{1140., 1222.}	63.3684	0.212998	newly 5 weeks spec	50.	87.25	{75., 99.}	74.5	0.239623	newly 5 weeks cumul	861.	1408.37	{1364., 1459.}	63.5738	0.213568	newly 52 weeks cumul	942.	1548.71	{1501., 1598.}	64.4066	0.215777
47	newly 3 weeks spec	224.	336.54	{313., 359.}	50.2411	0.176125	newly 3 weeks cumul	1089.	1622.55	{1573., 1680.}	48.9945	0.173039	newly 5 weeks spec	77.	147.66	{131., 166.}	91.7662	0.280922	newly 5 weeks cumul	1298.	1993.41	{1935., 2057.}	53.5755	0.186205	newly 47 weeks cumul	1446.	2271.57	{2197., 2332.}	57.0934	0.196045
48	newly 3 weeks spec	86.	151.77	{135., 170.}	76.4767	0.244881	newly 3 weeks cumul	493.	837.22	{806., 869.}	69.8215	0.229811	newly 5 weeks spec	18.	55.69	{45., 66.}	209.389	0.486371	newly 5 weeks cumul	561.	986.11	{948., 1020.}	75.7772	0.244786	newly 50 weeks cumul	581.	1068.66	{1020., 1114.}	83.9346	0.264462
49	newly 3 weeks spec	117.	230.6	{208., 251.}	97.094	0.293697	newly 3 weeks cumul	638.	1116.74	{1071., 1162.}	75.0376	0.242934	newly 5 weeks spec	40.	98.18	{86., 113.}	145.45	0.387393	newly 5 weeks cumul	753.	1364.42	{1312., 1414.}	81.1979	0.257991	newly 48 weeks cumul	828.	1547.62	{1488., 1595.}	86.9106	0.27149
50	newly 3 weeks spec	261.	239.32	{220., 260.}	8.61303	0.0398232	newly 3 weeks cumul	1311.	1192.89	{1157., 1228.}	9.00915	0.0411276	newly 5 weeks spec	104.	98.59	{88., 110.}	8.24038	0.0375018	newly 5 weeks cumul	1579.	1443.82	{1401., 1482.}	8.56111	0.0389709	newly 49 weeks cumul	1754.	1617.48	{1567., 1667.}	7.78335	0.035302
